# Age-Related Variations in the Population of Active Secondary Hair Follicles, Oxidative Stress and Antioxidant Parameters in Cashmere Goats

**DOI:** 10.3390/ani14091350

**Published:** 2024-04-30

**Authors:** Junxia Li, Zhenguo Wang, Xiayuan Wang, Jingxin Guo, Liujia Wang, Dong He, Xinming Duan, Chunxiang Zhang, Youshe Ren, Chunhe Yang

**Affiliations:** 1 College of Animal Science, Shanxi Agricultural University, Taigu 030801, China; lijunxia0121@163.com (J.L.); wangzhenguo_0059@163.com (Z.W.); wangxiayuan0404@163.com (X.W.); guo_jingxin@163.com (J.G.); 17332337209@163.com (L.W.); zhchx66@126.com (C.Z.); 2 Inner Mongolian Yiwei White Cashmere Goat Co., Ltd., Erdos 017000, China; 15149701806@163.com; 3 Nongfa Yuan (Hainan) Agricultural Development Co., Ltd., Haikou 570100, China; 17858795111@163.com; 4 Key Laboratory of Farm Animal Genetic Resources Exploration and Breeding of Shanxi Province, Taigu 030801, China

**Keywords:** cashmere goat, cashmere production, secondary hair follicle, age, oxidative stress

## Abstract

**Simple Summary:**

Previous research has demonstrated that the differences in yield, length, and fineness among different age groups are not proportional, indicating variations in cashmere density, specifically pertaining to the population of active secondary hair follicles. Results of this study revealed significant age-related variations in cashmere production (yield, staple length, and diameter) as well as the population of active secondary hair follicles among cashmere goats aged 2 to 7 years old. This study also revealed age-related variations in skin antioxidant capacity and oxidative damage in this age group. Specifically, the younger subgroup (aged 2–4 years old) exhibited significantly higher levels of cashmere production, population of active secondary hair follicles, and antioxidant capacity compared to those aged 5–7 years old. Furthermore, there were significant positive correlations between skin antioxidant capacity and the population of active secondary hair follicles. This study introduces a novel approach for enhancing the activity of secondary hair follicles and improving cashmere production through the regulation of oxidative stress.

**Abstract:**

The objective of this study was to investigate age-related changes in cashmere production and the population of active secondary hair follicles in cashmere goats across different age groups as well as to explore the association between secondary hair follicle activity and oxidative stress. A total of 104 adult Inner Mongolian ewe goats, aged between 2 and 7 years old, were randomly selected as experimental subjects. Skin samples were collected in August 2020 and cashmere samples were collected in April 2021. The cashmere fiber yield, staple length, and diameter showed age-related variations in cashmere goats aged 2 to 7 years (*p* < 0.05). Cashmere production was higher in goats aged 2–4 years compared to those aged 5–7 years (*p* < 0.05). There were no significant differences in the population of primary and secondary hair follicles among goats aged 2 to 7 years. However, the population of active secondary hair follicles varied significantly with age, with the younger group (aged 2–4 years) having a higher population than those aged 5–7 years (*p* < 0.05). A moderate negative correlation was observed between cashmere fiber diameter and the population of active secondary hair follicles (*p* < 0.05). Age-related variations in skin antioxidant capacity and oxidative damage were observed among cashmere goats aged 2 to 7 years old (*p* < 0.05). Goats aged 2 to 4 years exhibited higher antioxidant capacity and lower oxidative damage (*p* < 0.05). Interestingly, the skin’s antioxidant capacity and oxidative damage exhibited significant positive and negative correlations with the population of active secondary hair follicles (*p* < 0.05). This study presents a novel approach to enhance the activity of secondary hair follicles and improve cashmere production performance through the regulation of oxidative stress.

## 1. Introduction

The production of cashmere fiber, derived from secondary hair follicles in the skin of cashmere goats, is crucial in the textile industry and plays a pivotal role in manufacturing high-quality textiles. The quality and economic value of cashmere fiber primarily depend on its length and fineness, with particular emphasis on the latter. Various factors, including genetic factors [1] and non-genetic factors [2] such as nutrition, environment, sex, type of birth, age, etc., influence both the yield and quality of cashmere fiber. Therefore, it is of great significance to investigate these influencing factors to enhance both aspects.

The age-related variations in cashmere yield and quality have been extensively studied to assess individual lifetime productivity and optimize flock age structure for enhanced overall productivity. Significant variations were observed in the cashmere yield, length, and fineness of Inner Mongolian cashmere goats aged 1 to 8 years across different age groups [3,4]. A noticeable decline was observed from the age of 4 or 5 years old onwards, resulting in a substantial decrease in both quantity and quality [3,4]. Similarly, Shanbei white cashmere goats exhibited significant age-related variations in the yield and quality of their cashmere fiber, with a noticeable decline starting at the age of 4 years old [5,6]. The differences in cashmere yield, staple length, and fineness between 2-year-old and 5-year-old Shanbei white cashmere goats were found to be 17.47%, 20.00%, and 12.76%, respectively [5]. Similarly, the variations in cashmere yield, staple length, and fineness between 2-year-old and 6-year-old Inner Mongolian cashmere goats were observed to be 13.13%, 9.18%, and 3.12%, respectively [7]. The cashmere yield is determined by the length, fineness, and density of fiber. However, it should be noted that the variations in cashmere length, fineness, and yield among different age groups of cashmere goats are not directly proportional. This suggests differences in cashmere density, indicating varying populations of active secondary hair follicles. Additionally, previous studies have found a positive correlation between the population of secondary hair follicles in cashmere goats and cashmere yield as well as a negative correlation with cashmere fineness [8,9,10]. However, the relationship between the population of active secondary hair follicles in cashmere goats and cashmere fineness remains unknown. The investigation of the age-related changes in active secondary hair follicles and their correlation with cashmere fineness can provide a novel approach to enhancing cashmere production performance.

The maximum activity of secondary hair follicles in the skin of 1-year-old Inner Mongolian cashmere goats as well as Liaoning cashmere goats was reported to be 81% [11], 69% [12], and 70% [13], respectively. The peak activity of secondary hair follicles in Inner Mongolian cashmere goats aged 2–4 years was found to be 80% in our previous study [14]. Numerous studies have shown that excessive reactive oxygen species can cause oxidative stress-induced damage, leading to inhibited hair-follicle development and reduced follicle activity in both rodents and humans [15,16]. Previous studies have shown that exogenous melatonin can reactivate dormant secondary hair follicles, restoring their activity and increasing the population of active ones [14,17], indicating a potential relationship between oxidative stress and the activity of secondary hair follicles. However, the relationship between the activity of secondary hair follicles in cashmere goats and skin oxidative stress remains elusive. Therefore, we hypothesized that the activity of secondary hair follicles in cashmere goats undergoes age-related alterations and is correlated with oxidative stress. In this study, we conducted an analysis of age-related variations in cashmere production performance, including yield, length, and fineness, across different age groups of goats. Additionally, the investigation examined the population of active secondary hair follicles in goat skin along with the activity of skin antioxidant enzymes and oxidative stress damage. Furthermore, this study explored the relationship between the population of active secondary hair follicles and antioxidant enzyme activity as well as oxidative stress.

## 2. Materials and Methods

The experimental procedures were approved by the Institutional Animal Care and Use Committee of Shanxi Agricultural University (Taigu, China) under permission number SXAU-EAW-2021G. VQ.0080050143. The experiment was conducted at YiWei White Cashmere Goat Farm located in the Inner Mongolia Autonomous Region, Erdos, China (39°06′ N, 107°59′ E).

### 2.1. Animals, Experimental Design, and Management

A total of 104 adult ewe goats were randomly selected from a flock of 220 Inner Mongolian cashmere goats, including 16 two-year-olds, 16 three-year-olds, 18 four-year-olds, 20 five-year-olds, 17 six-year-olds and 17 seven-year-olds. The animals grazed in the pasture from 8 am to 5 pm and were fed in an open barn from 6 pm to 7 am year-round. The animals were bred in October, gave birth in March, and separated from their offspring in July. The animals were given additional supplementary concentrates from January to June to meet their nutrient requirements during gestation and lactation. The amount of supplementary feeding gradually increased from 0.275 kg/day per goat in January to 0.40 kg/day in April and further increased to 0.55 kg/day in May and June. The supplementary feed was obtained from Baotou Jiuzhoudadi Biotech Company (Baotou, China) and consisted of a 70% corn and 30% concentrate mixture.

### 2.2. Sample Collection

In August 2020, two skin biopsies were obtained from the right mid-side flank region using a 1 cm trephine, with the sampling positions of the two skins in close proximity to each other. The skin sample was placed onto tissue processing cassettes for tissue fixation, while another sample was frozen in liquid nitrogen and stored in a −80 °C freezer for tissue homogenization. After fixation in a 4% paraformaldehyde solution for 24 h, the skin samples were dehydrated using varying concentrations of ethanol and then embedded in paraffin for transverse sectioning. Fleece samples were collected from the left mid-side flank region of each goat within a 10 cm × 10 cm area to assess fiber staple length and diameter after shearing in April 2021. The cashmere weight of each goat was recorded following combing in April 2021.

### 2.3. Measurements of Cashmere Fiber Performance

The cashmere fibers were manually separated from fleece samples containing a mixture of coarse hairs and cashmere fibers before washing. They were then washed with carbon tetrachloride and distilled water, followed by air drying in a draught cupboard. The staple length of cashmere fibers was determined using the ruler method, following the established protocol outlined by Yang et al. [18]. The diameter of cashmere fibers was determined using an optical microscopic projection technique outlined by Peterson and Gherardi [19]. The trial methodologies are elucidated in our previously published article [20].

### 2.4. Calculations of Hair Follicle Population

**Figure 1:**
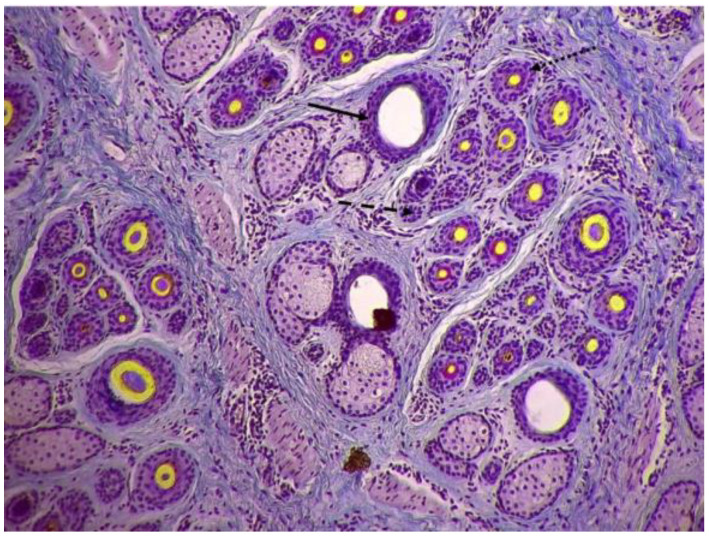
The typical structure of hair follicles in the skin of cashmere goats (10×). 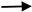 This symbol indicates the primary hair follicle. The primary follicles are characterized by the presence of hair follicles accompanied by sebaceous glands. 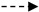 This symbol indicates the inactive secondary hair follicle. The inactive secondary follicles lacked a fiber canal or were filled with root sheath cells. 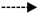 This symbol indicates the active secondary hair follicle. The active secondary follicles were defined as those with a fiber or a fiber canal, and a distinct red-stained inner root sheath surrounds the fiber.

The skin cross-sectional samples were prepared following the protocol described by Yang et al. [14]. The skin sections were stained using a modified Sacpic method, following Nixon’s protocol [21]. The precise methodology for quantifying hair follicles has been explicated in our previous publication [20]. The indicators of the population of primary hair follicles included primary follicle density index (PFDI) and number (PFN). The indicators of the population of secondary hair follicles included the secondary follicle density index (SFDI), number (SFN), and S:P ratio. Indicators related to the population of active secondary hair follicles included ASFDI (active secondary follicle density index), ASFN (active secondary follicle number), S*f*:P (ratio of active secondary follicles to primary follicles), and PASF (percentage of active secondary follicles). The typical structure of hair follicles in the skin of cashmere goats is depicted in Figure 1.

### 2.5. Statistical Analysis

The statistical analyses were conducted using SAS 9.2 software from SAS Institute Inc. (Cary, NC, USA). The cashmere production performance of cashmere goats aged 2 to 7 years old, including yield, staple length, and diameter, was analyzed using a one-way ANOVA. Similarly, various indicators related to primary hair follicle population (PFDI, PFN), secondary hair follicle population (SFDI, SFN, S:P), active secondary hair follicle population (ASFDI, ASFN, S*f*:P, PASF) as well as skin antioxidant capacity and oxidative damage were evaluated using a one-way ANOVA. The relationship between cashmere fiber diameter and indicators associated with primary hair follicle population (PFDI, PFN), secondary hair follicle population (SFDI, SFN, S:P), and active secondary hair follicle population (ASFDI, ASFN, S*f*:P, PASF) was examined using the CORR procedure of the Pearson correlation analysis. Additionally, a Pearson correlation analysis was performed to investigate the relationship between indicators of active secondary hair follicle population and antioxidant capacity as well as oxidative damage. Correlation coefficients were interpreted based on these criteria: |r| ≥ 0.5 indicated a strong correlation; 0.3 ≤ |r| < 0.5 indicated a moderate correlation; 0.1 ≤ |r| < 0.3 indicated a weak correlation; and |r| < 0.1 indicated no correlation [22]. The data are presented as mean ± standard deviation (mean ± SD). Statistical significance was set at *p* < 0.05 for significance and *p* < 0.01 for extreme significance.

## 3. Results

### 3.1. Cashmere Fiber Yield, Staple Length, and Diameter in Cashmere Goats Aged 2 to 7 Years Old

**Figure 2:**
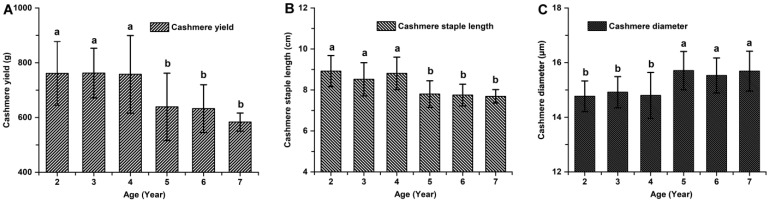
Cashmere production performance in cashmere goats aged 2 to 7 years old. (**A**) cashmere yield. (**B**) cashmere staple length. (**C**) cashmere diameter. No significant differences (*p* > 0.05) between groups are indicated by marking the same letter a or b, while the significant differences (*p* < 0.05) are indicated by marking different letters a and b.

The cashmere fiber yield, staple length, and diameter in cashmere goats aged 2 to 7 years old exhibited significant age-related variations (*p* < 0.05; Figure 2). There was no statistically significant difference observed in the cashmere production performance of 2–4-year-old goats (*p* > 0.05; Figure 2). Similarly, the cashmere production performance of 5–7-year-old goats did not show any statistically significant difference (*p* > 0.05; Figure 2). However, there was a significant difference between the cashmere production performance of goats aged 2–4 years and those aged 5–7 years (*p* < 0.05). The cashmere yield of 2–4-year-old goats was found to be higher by 23.23% (760.7 ± 108.6 g vs. 617.3 ± 88.1 g), while their staple length was observed to be longer by 15.10% (8.92 ± 1.00 cm vs. 7.75 ± 0.51 cm) compared to that of the older ones aged 5–7 years old, respectively. The cashmere diameter of younger goats (aged 2–4 years old) decreased by about 4.61% compared with that of older ones (aged 5–7 years old) (14.91 ± 0.72 μm vs. 15.63 ± 0.80 μm).

### 3.2. Population of Active Secondary Hair Follicles in Cashmere Goats Aged 2 to 7 Years Old

**Figure 3:**
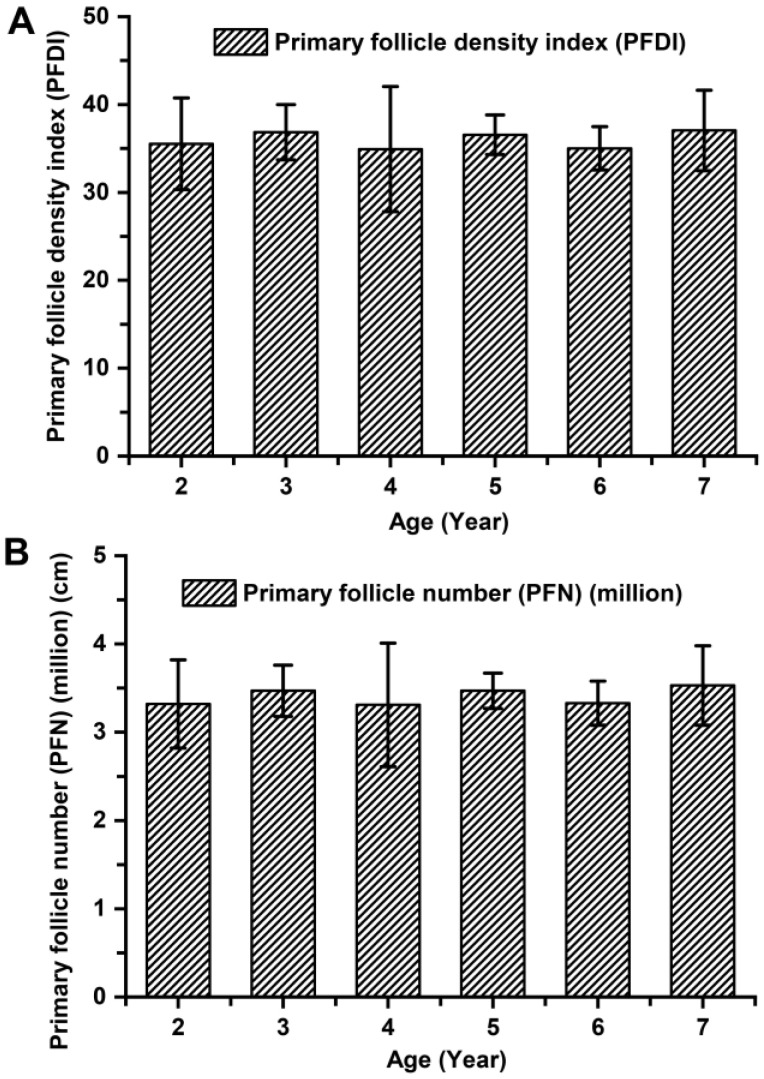
The population of primary hair follicles among cashmere goats aged 2 to 7 years. (**A**) Primary follicle density index among cashmere goats aged 2 to 7 years. (**B**) Primary follicle number among cashmere goats aged 2 to 7 years.

**Figure 4:**
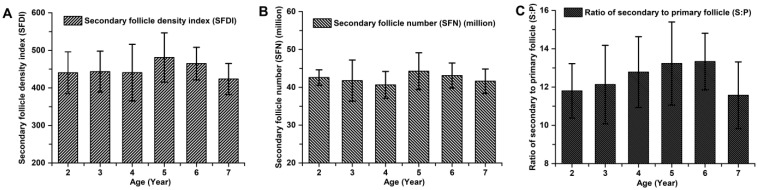
The population of secondary hair follicles among cashmere goats aged 2 to 7 years. (**A**) Secondary follicle density index among cashmere goats aged 2 to 7 years. (**B**) Secondary follicle number among cashmere goats aged 2 to 7 years. (**C**) Ratio of secondary to primary follicle among cashmere goats aged 2 to 7 years.

**Figure 5:**
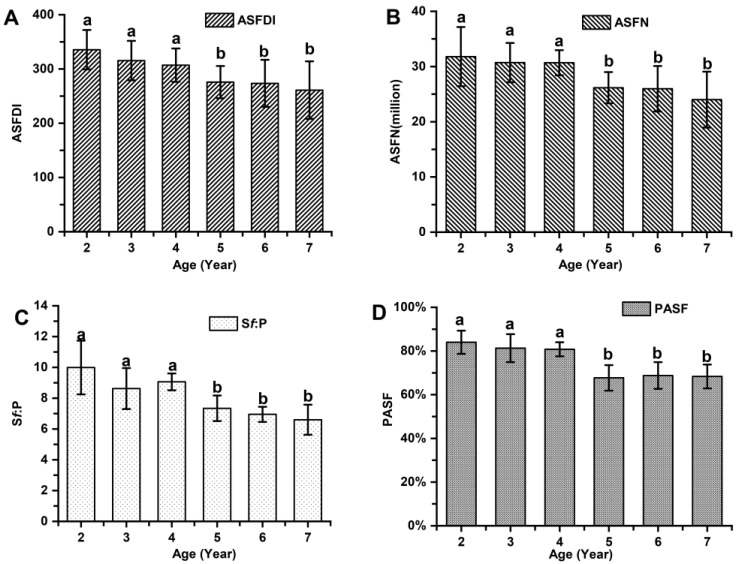
The population of active secondary hair follicles among cashmere goats aged 2 to 7 years. (**A**) Active secondary follicle density index among cashmere goats aged 2 to 7 years. (**B**) Active secondary follicle number among cashmere goats aged 2 to 7 years. (**C**) Ratio of active secondary to primary follicle among cashmere goats aged 2 to 7 years. (**D**) Percentage of active secondary follicles among cashmere goats aged 2 to 7 years. No significant differences (*p* > 0.05) between groups are indicated by marking the same letter a or b, while the significant differences (*p* < 0.05) are indicated by marking different letters a and b.

The indicators associated with the primary hair follicle population (PFDI and PFN), showed no significant differences among cashmere goats aged 2 to 7 years old (*p* > 0.05; Figure 3). The average values of PFDI and PFN in cashmere goats aged 2 to 7 years old were recorded as 35.87 ± 4.36 and 3.39 ± 0.43, respectively. Similarly, there were no significant differences observed in the indicators associated with the secondary hair follicle population (SFDI, SFN, and S:P) (*p* > 0.05; Figure 4). Additionally, the mean values of SFDI, SFN, and S:P in cashmere goats aged between 2 to 7 years old were determined as follows: SFDI, 449.35 ± 56.50; SFN, (42.31 ± 3.67) million; S:P, 12.55 ± 1.80. The indicators associated with the active secondary hair follicle population, including ASFDI, ASFN, PASF, and S*f*:P, showed significant variations (*p* < 0.05; Figure 5). The ASFDI of cashmere goats aged 2–4 years was found to be 18.23% higher (319.35 ± 34.46 vs. 270.10 ± 41.99) compared to that of cashmere goats aged 5–7 years (*p* < 0.05). Additionally, the younger age group (aged 2–4 years old) exhibited a significantly higher ASFN and PASF by 22.38% (31.06 ± 3.73 vs. 25.38 ± 4.01) and by 20.15% (82.04 ± 5.00 vs. 68.28 ± 5.81), respectively (*p* < 0.05). Furthermore, the S*f*:P ratio in cashmere goats aged 2–4 years was 32.47% higher (9.22 ± 1.21 vs. 6.96 ± 0.77) compared to those aged 5–7 years (*p* < 0.05).

### 3.3. Relationship between Population of Primary and Secondary Hair Follicles and Cashmere Fiber Diameter in Cashmere Goats Aged 2 to 7 Years

In the present study, there were no significant correlations between cashmere fiber diameter and indicators of primary hair follicle population (PFDI and PFN) in cashmere goats aged 2 to 7 years old (*p* > 0.05; Table 1). However, a significant negative moderate correlation was found between cashmere fiber diameter and indicators of secondary hair follicle population (SFDI, SFN, and S:P) in the same age group of cashmere goats (*p* < 0.05; Table 1). Additionally, a significant negative moderate correlation was observed between cashmere fiber diameter and indicators of active secondary hair follicle population (ASFDI, ASFN, and S*f*:P) in the same age group of cashmere goats (*p* < 0.05; Table 1).

### 3.4. Skin Antioxidant Capacity and Oxidative Damage in Cashmere Goats Aged 2–7 Years Old

**Figure 6:**
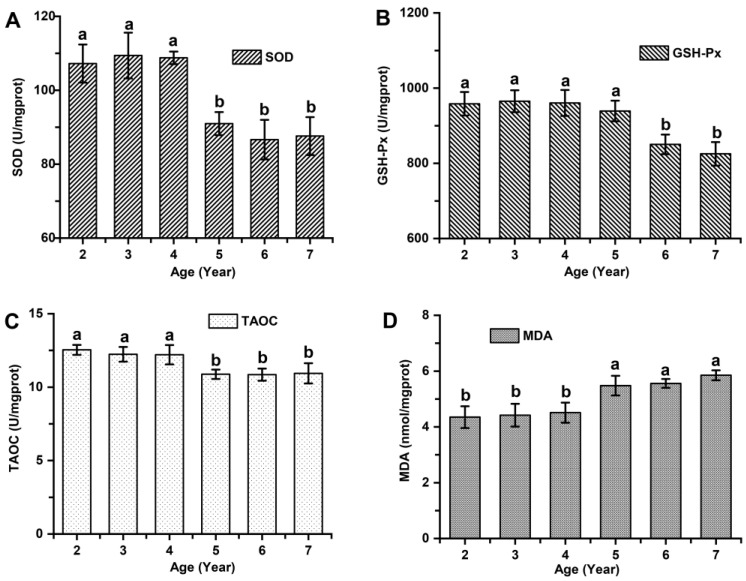
Skin antioxidant capacity and oxidative damage among cashmere goats aged 2 to 7 years old. (**A**) The activity of SOD enzyme among cashmere goats aged 2 to 7 years old. (**B**) The activity of GSH-Px enzyme among cashmere goats aged 2 to 7 years old. (**C**) The level of TAOC among cashmere goats aged 2 to 7 years old. (**D**) The content of MDA among cashmere goats aged 2 to 7 years old. No significant differences (*p* > 0.05) between groups are indicated by marking the same letter a or b, while the significant differences (*p* < 0.05) are indicated by marking different letters a and b.

Significant variations in skin antioxidant capacity were observed among cashmere goats aged 2 to 7 years old (*p* < 0.05; Figure 6). Cashmere goats aged 2 to 4 years exhibited significantly higher levels of superoxide dismutase (SOD) by 22.87% (108.53 ± 4.87 vs. 88.33 ± 4.76), glutathione peroxidase (GSH-Px) by 10.29% (961.23 ± 31.77 vs. 871.52 ± 28.37), and total antioxidant capacity (TAOC) by 14.34% (12.20 ± 0.54 vs. 10.67 ± 0.92) compared to those aged 5 to 7 years (*p* < 0.05). Furthermore, significant differences in oxidative damage were found among cashmere goats aged 2 to 7 years old, with the younger age group (2 to 4 years old) showing a remarkable decrease of MDA levels—a biomarker reflecting oxidative stress-induced damage by approximately 16.73% (4.38 ± 0.41 vs. 5.26 ± 0.65) compared to the older age group (5 to 7 years old) (*p* < 0.05).

### 3.5. Association between Antioxidant Capacity and Active Secondary Hair Follicle Population in Cashmere Goats Aged 2 to 7 Years Old

The SOD activity showed significant positive correlations with indicators of active secondary hair follicle population, including ASFDI, ASFN, and S*f*:P ratio (*p* < 0.05; Table 2). There was a trend indicating a moderate positive correlation between SOD activity and PASF (*p* = 0.051; Table 2). Additionally, the GSH-Px activity showed significant positive correlations with indicators of active secondary hair follicle population such as ASFDI, ASFN, PASF, and S*f*:P ratio (*p* < 0.05; Table 2). Moreover, the TAOC levels demonstrated significant positive correlations with indicators of active secondary hair follicle population like ASFDI, ASFN, and S*f*:P ratio (*p* < 0.05; Table 2). Similarly, there were significant negative correlations between MDA content and indicators of active secondary hair follicle population, including ASFDI, ASFN, PASF, and S*f*:P ratio (*p* < 0.05; Table 2).

## 4. Discussion

Previous studies have demonstrated significant variations in cashmere yield, staple length, and fineness among cashmere goats of different ages, indicating differences in cashmere density due to the population of active secondary hair follicles in the skin [3,7]. Additionally, research on hair follicle development and growth in mice and humans has demonstrated that the excessive generation of reactive oxygen species (ROS), leading to oxidative stress, plays a crucial role in the progression of hair follicle atrophy, diminished activity, and eventual degeneration [16,23,24]. However, the correlation between secondary hair follicle activity in cashmere goats of different ages and antioxidant capacity as well as oxidative stress damage remains unknown. The present study reveals variations in cashmere production performance among different age groups of cashmere goats, with significantly higher performance observed in 2–4-year-old goats compared to those aged 5–7 years. Similar alterations were found across different age groups regarding the population of active secondary hair follicles in their skin. Age-dependent changes were observed for antioxidant enzyme activity with significantly higher levels detected in 2–4-year-old goats compared to those aged 5–7 years. Conversely, age-related changes were observed for MDA levels exhibiting an inverse pattern. Importantly, correlation analysis results revealed a significant negative correlation between secondary hair follicle activity and skin oxidative stress damage.

The findings of previous studies have demonstrated age-related variations in cashmere yield, staple length, and fineness among cashmere goats of varying ages. The staple length of cashmere fiber in Longdong cashmere goats aged 2–4 years was found to be significantly higher compared to those aged 5–7 years [25]. Similarly, the present study found that the staple length of cashmere fiber in Inner Mongolian cashmere goats aged 2–4 years was 15.10% higher than those aged 5–7 years. Previous studies have demonstrated a significant increase in the cashmere yield of Inner Mongolian cashmere goats aged 2–4 years [3], Shanbei white cashmere goats aged 2–4 years [26], and Longdong cashmere goats aged 2–4 years [25] compared to those aged 5–7 years, while there has been a notable decrease in the diameter of the cashmere fiber. The present study revealed a comparable increase in cashmere yield, with the cashmere yield of Inner Mongolian cashmere goats aged 2–4 years being 23.23% higher than that of the goats aged 5–7 years. The present study, however, found that the cashmere diameter in Inner Mongolian cashmere goats aged 5–7 years was 4.61% higher than that of goats aged 2–4 years. The diameter of cashmere fibers in Inner Mongolian cashmere goats was reported to undergo a significant increase beyond the age of 5 years [7]. The inconsistent findings regarding the variation in cashmere diameter of cashmere goats at different ages may be attributed to the nutrient level during the period of secondary hair follicle reconstruction. Our previous research showed that cashmere goats with high body weight gain during the period of secondary hair follicle reconstitution exhibited a greater cashmere diameter compared to those with low body weight gain [20]. The cashmere yield depends on the length, diameter, and density of the fiber; however, these factors do not exhibit a proportional relationship across different ages of cashmere goats. This implies that variations in the population of active secondary hair follicles within the skin contribute to disparities in cashmere density among different age groups.

The morphogenesis of secondary hair follicles in the skin of cashmere goats has been confirmed to commence during the fetal stage and reach maturity at 3–6 months postpartum [18,27,28]. Upon reaching maturity, the population of secondary hair follicles remains constant, subsequently undergoing a cyclical process comprising catagen, telogen, and anagen [12,14]. It was reported that the skin S:P ratio remained consistent across different age groups (6 months, 1 year, and 2 years) for both Longdong [29] and Inner Mongolian cashmere goats aged 1–3 years old [27], suggesting a stable population of secondary hair follicles. In addition, the skin S:P ratio of one-year-old Inner Mongolian and Liaoning cashmere goats remains consistent throughout the year without significant variation [13]. In the present study, no statistically significant variations were observed in the population of secondary hair follicles in the skin of cashmere goats across different age groups, as indicated by SFDI, SFN, and S:P. This finding further validates the precision of the slicing technique in this study and establishes a solid foundation for quantitative assessment of active secondary hair follicles. The activity of secondary hair follicles in the skin of cashmere goats has been confirmed to peak during the months of August and September [11,12,30]. The activity of secondary hair follicles in the skin of one-year-old Inner Mongolian cashmere goats as well as Liaoning cashmere goats was reported to be 81% [11], 69% [12], and 70% [13], respectively. In the present study, the activity of secondary hair follicles in the skin of Inner Mongolian cashmere goats aged 2–7 years ranged from 67.70% to 84.02%. Importantly, the findings of this study revealed significant age-related variations in the population of active secondary hair follicles, as indicated by ASFDI, ASFN, PASF, and S*f*:P ratio. The population of active secondary hair follicles in goats aged 2–4 years was significantly higher compared to those aged 5–7 years. The findings of this study demonstrate that not all secondary hair follicles in cashmere goats undergo a transition from telogen to anagen, with some secondary hair follicles undergoing atresia, thereby resulting in reduced activity of secondary hair follicles and cashmere yield.

The fineness of cashmere fiber plays a crucial role in determining both the quality and economic value of cashmere. Previous studies have confirmed a negative correlation between the fineness of cashmere fiber and the population of secondary hair follicles in the skin of cashmere goats [9]. Increasing the population of secondary hair follicles is an important approach to reducing the fineness of cashmere fiber [17,18]. The fineness of cashmere fiber exhibited a significant negative correlation with the skin S:P ratio in 1-year-old Shanbei white cashmere goats (r = −0.434) [10] and 6-month-old Inner Mongolian cashmere goats (r = −0.488) [31]. Similarly, a negative correlation (r = −0.330) was observed between the fineness of cashmere fiber and the skin S:P ratio in Inner Mongolian cashmere goats aged 2–7 years in this study. Furthermore, the secondary hair follicle population indicators in Inner Mongolian cashmere goats aged 2–7 years, such as SFDI and SFN, exhibited a negative correlation with the fineness of cashmere fiber (r = −0.374 and r = −0.348, respectively). Our findings provide further evidence of a negative correlation between the population of secondary hair follicles and the fineness of cashmere fiber, irrespective of the age of cashmere goats. Interestingly, the correlation analysis results of the present study revealed a significant moderate negative correlation between the population of active secondary hair follicles and cashmere fiber diameter. The findings of the present study suggest that the variation in active secondary hair follicle populations among cashmere goats of different ages contributes partially to variations in cashmere yield, rather than differences in the overall populations of secondary hair follicles. In addition, the findings suggest that, in addition to increasing the population of total secondary hair follicles, enhancing the metabolic activity of secondary hair follicles can increase cashmere yield in individual cashmere goats. Therefore, it is crucial to elucidate the underlying mechanisms of age-related atresia and decline in secondary hair follicle activity in order to implement interventions aimed at enhancing secondary hair follicle activity and cashmere performance.

The hair follicles are frequently exposed to high levels of reactive oxygen species (ROS), causing oxidative stress and subsequent apoptosis in the progenitor cells and dermal papilla cells, ultimately leading to structural impairment [15]. Previous studies demonstrated that the function of reactive oxygen species (ROS) in hair follicles is dualistic: a low level of ROS can stimulate the transition from telogen to anagen, while a high level of ROS can hinder this process and induce regression of hair follicles [23]. The excessive production of reactive oxygen species (ROS) induced by obesity can initiate the activation of inflammatory signals and abnormal accumulation of ROS and lipids in murine hair follicle stem cells, thereby impairing the metabolic activity of hair follicles [32]. During the transition from telogen to early anagen stage, dermal papilla cells release signals that stimulate metabolic activity in hair progenitor cells, promoting proliferation and generating reactive oxygen species (ROS); failure to promptly eliminate these ROS can lead to oxidative stress [16,33]. In the present study, the antioxidant enzyme activity in the skin of 2–4-year-old cashmere goats was significantly higher than that observed in 5–7-year-old goats, while the MDA content showed a significant decrease compared to the latter group. The findings of this study align with those observed in rodent models, including rats and mice as well as in avian species such as laying hens. The activity of serum SOD decreased significantly in aged rats compared to young rats, while the MDA content increased significantly [34]. The activity of the SOD enzyme in the intervertebral disc homogenate [35] and brain [36] decreased significantly in aged mice compared to the young and adult groups, while the concentration of MDA increased significantly. The activities of the SOD enzyme and CAT enzyme in testicular tissue gradually declined from 8 months of age in mice [37]. During the late laying period, there was a notable decrease in laying performance along with decreased levels of GSH content, T-AOC, total superoxide dismutase (SOD), catalase (CAT), and glutathione S-transferase (GST) in ovarian tissue of hens [38]. Conversely, MDA content as well as hydrogen peroxide and ROS levels exhibited a significant increase when compared to the initial and peak laying periods [39]. In the present study, the changes in antioxidant enzyme activity and oxidative stress injury in the skin of cashmere goats at different ages were consistent with variations in active secondary hair follicles. The results of correlation analysis revealed significant positive correlations between the indicators of active secondary hair follicle population (ASFDI, ASFN, and S*f*:P ratio) and skin antioxidant enzyme activity. Additionally, these indicators showed notable negative associations with skin malondialdehyde (MDA) content. Our findings suggest a preliminary correlation between oxidative stress and diminished activity of secondary hair follicles in cashmere goats. However, the relationship between oxidative stress and the activity of secondary hair follicles in the skin of cashmere goats, as well as the underlying mechanism, requires further investigation.

## 5. Conclusions

The cashmere fiber yield, staple length, and diameter showed significant age-related variations in cashmere goats aged 2 to 7 years. The population of active secondary hair follicles and skin antioxidant capacity exhibited significant age-related variations among cashmere goats aged 2 to 7 years old, with the younger group (aged 2–4 years) having a significantly higher level than those aged 5–7 years. Importantly, antioxidant capacity and oxidative damage exhibited significant positive and negative correlations, respectively, with the population of active secondary hair follicles. This study presents a novel approach to enhancing the activity of secondary hair follicles and improving cashmere production performance through the regulation of oxidative stress. Further investigation is required to explore the relationship between oxidative stress and the activity of secondary hair follicles in the skin of cashmere goats as well as the underlying mechanism.

## Figures and Tables

**Table 1:** The correlation coefficient among the population of primary follicles, secondary follicles, active secondary follicles, and cashmere diameter.

Item	PFDI	PFN	SFDI	SFN	S:P	ASFDI	ASFN	S*f*:P	PASF
Cashmere fiber diameter	0.164	0.155	−0.374	−0.348	−0.330	−0.376	−0.328	−0.370	−0.397
*p*-value	0.283	0.310	0.023	0.032	0.033	0.018	0.036	0.024	0.010

**Table 2:** The correlation coefficient between the population of active secondary follicles and skin antioxidant capacity and oxidative damage among cashmere goats aged 2 to 7 years old.

Item	ASFDI	ASFN	PASF	S*f*:P
SOD	0.462(0.030)	0.432(0.045)	0.421(0.051)	0.594(0.020)
GSH-Px	0.462(0.030)	0.434(0.044)	0.456(0.033)	0.555(0.039)
TAOC	0.412(0.045)	0.489(0.040)	0.378(0.069)	0.556(0.039)
MDA	−0.510(0.013)	−0.490(0.018)	−0.445(0.033)	−0.575(0.010)

Note: *p*-values are represented in parentheses.

## Data Availability

Data is contained within the article.
